# Differential responses to fertilization and competition among invasive, noninvasive alien, and native *Bidens* species

**DOI:** 10.1002/ece3.7071

**Published:** 2020-11-24

**Authors:** Sunghyun Woo, Dongyeob Lee, Yong‐Chan Cho, Sangsun Lee, Eunsuk Kim

**Affiliations:** ^1^ School of Earth Sciences and Environmental Engineering Gwangju Institute of Science and Technology Gwangju Korea; ^2^ Korea National Arboretum Pocheon Korea

**Keywords:** functional traits, invasive species, phenotypic plasticity, seed morphology, trait differences

## Abstract

Comparative studies of invasive, noninvasive alien, and native congenic plant species can identify plant traits that drive invasiveness. In particular, functional traits associated with rapid growth rate and high fecundity likely facilitate invasive success. As such traits often exhibit high phenotypic plasticity, characterizing plastic responses to anthropogenic environmental changes such as eutrophication and disturbance is important for predicting the invasive success of alien plant species in the future. Here, we compared trait expression and phenotypic plasticity at the species level among invasive, noninvasive alien, and native *Bidens* species. Plants were grown under nutrient addition and competition treatments, and their functional, morphological, and seed traits were examined. Invasive *B. frondosa* exhibited higher phenotypic plasticity in most measured traits than did the alien noninvasive *B. pilosa* or native *B. bipinnata*. However, differential plastic responses to environmental treatments rarely altered the rank of trait values among the three *Bidens* species, except for the number of inflorescences. The achene size of *B. frondosa* was larger, but its pappus length was shorter than that of *B. pilosa*. Two species demonstrated opposite plastic responses of pappus length to fertilization. These results suggest that the plasticity of functional traits does not significantly contribute to the invasive success of *B. frondosa*. The dispersal efficiency of *B. frondosa* is expected to be lower than that of *B. pilosa*, suggesting that long‐distance dispersal is likely not a critical factor in determining invasive success.

## Graphical abstract

We conducted a comparative study examining phenotype expression of invasive, noninvasive alien, and native *Bidens* species under fertilization and competition treatment. While invasive *B. frondosa* exhibited higher plasticity than other species, the plasticity of functional traits does not likely contribute to the invasive success. The dispersal efficiency of *B. frondosa* is expected to be lower than that of other *Bidens* species, suggesting that long‐distance dispersal is likely not a critical factor in determining invasive success.

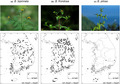

## 1 INTRODUCTION

Invasive plant species have been hypothesized to possess a suite of traits associated with successful invasion into areas in which they are introduced (van Kleunen et al., 2010; Rejmánek & Richardson, 1996). For instance, invasive species often have functional traits that confer greater resource capture ability, such as larger specific leaf areas (SLA) and smaller root‐to‐shoot ratios (R/S ratios) (Ehrenfeld, 2003; Grotkopp & Rejmánek, 2007). These traits result in faster growth rates and higher reproductive outputs of invasive species than those in native or noninvasive alien species (Mathakutha et al., 2019). Although the contribution of such traits to invasive success depends on the ecological context (Catford et al., 2009), an investigation of trait variations between invasive and noninvasive plants is a first step in identifying candidate invasive alien species for weed‐risk assessments and in formulating an trait‐based predictions to invasiveness (Gallagher et al., 2014; van Kleunen et al., 2015; Mathakutha et al., 2019).

The comparison between native and invasive plant species is a widely adopted method to infer traits that determine invasive success. However, the information gleaned often seems inadequate to evaluate attributes of invasive species (van Kleunen et al., 2015). First, the choice of a target native species is critical, because a comparison between invasive and co‐occurring native species with different life cycles is likely to yield misleading results (Daehler, 2003; Vilá & Weiner, 2004). In addition, a comparison between invasive and noninvasive alien species would be more appropriate to identify traits contributing to invasive success (van Kleunen, Dawson, et al., 2010). A comparative study considering invasive, noninvasive alien, and native plant species, all of which are congenic, is required to gain insight into the traits that determine invasiveness.

Traits associated with resource capture ability are highly plastic to environmental conditions, particularly resource availability (Davidson et al., 2011; Funk, 2008; Gioria & Osborne, 2014; Godoy et al., 2012). Invasive plants tend to exhibit higher phenotypic plasticity than noninvasive plants, contributing to their establishment in novel environments and competition with existing vegetation (Davidson et al., 2011). Given the ecological significance of plasticity in invasive success, phenotypic plasticity to anthropogenic environmental change is of particular interest for the prediction of the future dynamics of plant invasion. Human activities continue to increase disturbance and eutrophication in terrestrial habitats, both of which are thought to facilitate the invasion of alien plant species (Chytrý et al., 2008; Davis et al., 2000; Jauni et al., 2015). Disturbance removes competing species, consequently decreasing biotic interactions between species while increasing soil nutrient availability. Though plant responses to nutrient availability are widely studied, little information is available on phenotypic plasticity to environmental disturbance (Gioria & Osborne, 2014).

Since plants are sessile organisms, seed dispersal and germination are critical life stages for habitat selection and thereby influence species range (Donohue et al., 2010). A widely held hypothesis suggests that invasive plants tend to have smaller fruits or seeds than native species, thus facilitating seed dispersal and rapid range expansion (Coutts et al., 2011; Rejmánek & Richardson, 1996). However, diverse fruit traits other than seed size can affect dispersal efficiency (Römermann et al., 2005), and these traits could be plastic to environmental conditions, as are other morphological and functional traits. Given the ecological significance of dispersal strategies, fruit characteristics, and their plasticities in plant invasiveness, these characteristics should be evaluated more extensively (Doudová et al., 2017).

**FIGURE 1:**
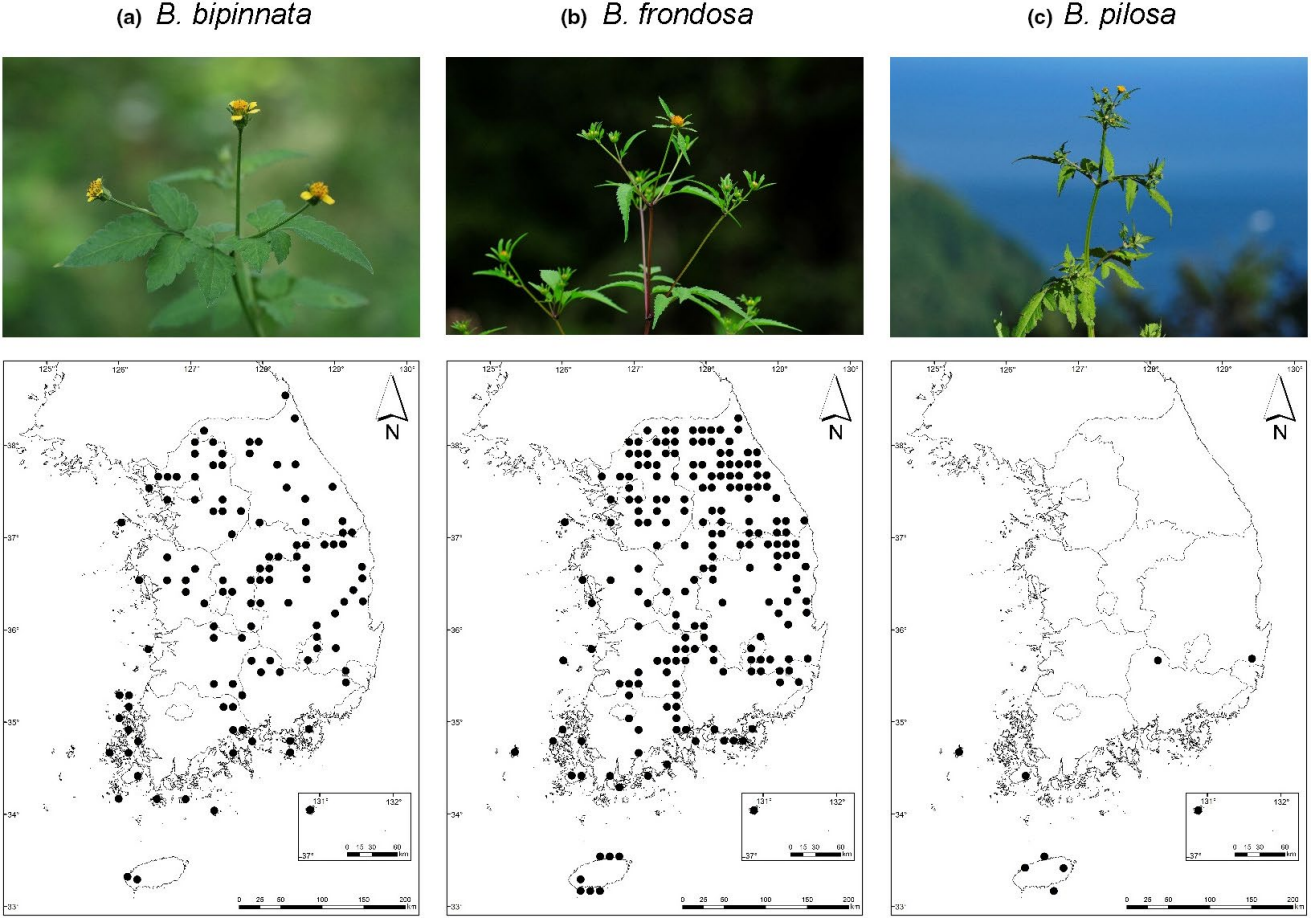
Photographs of testing plant species and their distribution in South Korea (Korea National Arboretum, 2016)

*Bidens bipinnata* is native to East Asia (Wang et al., 2017). *B. frondosa,* which originated in North America, and *B. pilosa*, originated in tropical America, have been reported in the Korean Peninsula since 1964 (Park et al., 2002). *B. frondosa* occurs throughout South Korea, but *B. pilosa* is mainly found near the coastal area, especially around major harbors of South Korea (Figure 1) (Korea National Arboretum, 2016). We treated *B. pilosa* as a noninvasive alien in this study because its distribution is restricted to the candidate origin of introduction despite the maintenance of natural populations (Blackburn et al., 2011; Mathakutha et al., 2019). Achenes of *Bidens* species have pappi that consist of two to three barbed awns that facilitate adhesive dispersal (Sorensen, 1986). Since plant species with smaller seed sizes or longer awns tend to have a higher probability of remaining attached to animal fur (Ansong & Pickering, 2014; Kiviniemi & Telenius, 1998), both achene size and pappus length in *Bidens* species likely affect dispersal efficiency (Rocha, 1996).

Phenotypic plasticity is defined as the differential phenotypic expression of a genotype in response to environmental factors, so an experimental study using clonal replicates is ideal for examining phenotypic plasticity (Pigliucci, 2001). However, ecological studies often evaluate plasticity at the population or species level by comparing the average trait value of a population or species between environmental treatments (Davidson et al., 2011; Richards et al., 2006). Although the results might confound plasticity with genotypic variation between treatments, evaluation of phenotypic plasticity at the species level has an advantage of including diverse genotypes representing a species. If there is no bias in assigning individual plants to environmental treatments, plasticity at the species level can provide information on the ecological significance of plasticity in invasive species (Davidson et al., 2011; Richards et al., 2006).

Here, we examined the phenotypic expression of native and alien *Bidens* species in response to nutrient availability and disturbance. Specifically, the following questions were addressed: (a) Do native, noninvasive, and invasive alien *Bidens* species exhibit differential morphological and functional traits? (b) Do *Bidens* species respond differently to fertilization and competition treatments? (c) Does invasive *Bidens* species have morphological characteristics that better facilitate long‐distance dispersal compared to noninvasive species?

## 2 MATERIALS AND METHODS

### 2.1 Study species and seed sources

We examined two alien (*B. frondosa*, *B. pilosa*) and one native *Bidens* species (*B. bipinnata*), Asteraceae. All three species are herbaceous erect weedy species that inhabit disturbed areas or the periphery of rice fields in Korea. The achenes of *B. bipinnata* and *B. pilosa* are oblong‐shaped with three to four barbed awns, but that of *B. frondosa* is obovate‐shaped with two awns. The achenes used in this study were obtained from the seed stock center in the Korean National Arboretum. According to the information of the seed stock center, achenes of *B. bipinnata* and *B. frondosa* were collected from natural populations in Pocheon‐si (37° 46′ 20.057″ N, 127° 9′ 56.314″ E), and *B. pilosa* were collected in Seogwipo‐si, Korea (33° 14′ 43.062″ N, 126° 24′ 40.24″ E) in 2014. Since we used field‐collected achenes for this study, maternal effects might influence the results. However, our primary objective is comparing trait expressions between species, and maternal effects likely have limited effects on the species‐level comparisons.

### 2.2 Experimental design

Achenes of *Bidens* species were sterilized using 0.5% sodium hypochlorite for 10 min, carefully scarified twice with a razor blade, and maintained in Petri dishes with wet filter paper in June 2016. After cold treatment at 4°C for 2 weeks, achenes were allowed to germinate in a customized walk‐in chamber under a 12‐hr light/dark photoperiod at 23°C. Individual seedlings were planted in flats containing commercial soil medium (ShinSung Mineral Co. Ltd.) and grown under the same conditions present during germination. After one month of growth, plants were transplanted into plastic pots (21.5 cm × 21.4 cm × 15.5 cm) filled with 50% commercial saprolite and 50% soil medium on August 25, 2016. The plants were maintained in a common garden at the Gwangju Institute of Science and Technology (35°13′28.47″ N, 126°50′36.81″ E). According to the Korean Meteorological Administration, the temperature during the 2016 summer season was the second hottest since 1973, and precipitation was around half of the ten‐year average. To prevent transplant shock and the abrupt death of plants, plants were watered every other day for one month.

To evaluate the effects of competition and fertilization, we employed a full factorial design with four treatment combinations. As a competition treatment, we transplanted one individual seedling of each *Bidens* species to the center of pots containing *Festuca arundinacea* (tall fescue). *F. arundinacea* was chosen as a competing species because it has been widely used to recover anthropogenically disturbed areas in Korea. Before transplantation, 1 g of *F. arundinacea* seeds was sowed in each pot following the distributor's recommendations and grown for one and a half months in the common garden. To simulate the high‐nutrient content of soil in agricultural areas, half of the pots in each competition treatment received 1 g of slow‐release fertilizer pellets (Osmocote Plus N:P:K 13 – 13 – 13 + 2MgO + trace element, Everris International B.V.) following the manufacturer's instructions. Pots containing plants were randomly positioned using a completely randomized design. Thirty individuals of each plant species were used for each of the four treatments, resulting in a total of 360 plants. We harvested half of the plants to measure plant growth and functional traits 6 weeks after the treatment application. The remaining plants were grown until they completed reproduction to measure fecundity and achene traits.

### 2.3 Trait measurement

After 6 weeks of treatment, we collected whole plants, washed their roots to remove all soil, and dried them in a dry oven (Hanbaek, Co. Ltd.) at 65°C for three days to weigh their dry mass. The R/S ratio was calculated as the dry mass of the root divided by the aboveground biomass. Before harvesting, we took one fully expanded leaf from each plant and scanned it to measure the leaf area (Digimizer software ver. 4.6.1; MedCalc Software bvda). Leaves were dried at 65°C for 3 days, and the SLA was calculated as the leaf area divided by the dry mass. We estimated the leaf chlorophyll content using a SPAD‐520 plus chlorophyll meter (Spectrum Technologies). The SPAD values exponentially correlate with the chlorophyll content.

We counted the number of inflorescences as a proxy of fecundity, because many fruits fell onto the ground during the experiment. *Bidens* species produce morphologically distinctive achenes at the center and periphery of the capitulum (Brändel, 2004). Disk achenes tend to be larger and disperse more readily than ray achenes (Rocha, 1996). We randomly selected more than two achenes from the central area of the capitulum of each plant individual and photographed them using a stereomicroscope. Images were analyzed using Digimizer software to measure the length of the pappi and the cross‐sectional areas of the achenes. Achenes with broken pappi were excluded from the image analysis, and morphological characteristics were analyzed for a total of 250 *B. bipinnata* achenes, 290 *B. frondosa* achenes, and 140 *B. Pilosa* achenes.

### 2.4 Statistical analyses

In order to evaluate the phenotypic plasticity of *Bidens* species, we computed the relative distance plasticity index (RDPI) of each trait following Valladares et al. (2006). For each species, we calculated relative distances of trait values between all pairs of individuals that were grown in different environments. The RDPI for the competition was the average of the relative distances between competition treatments in the absence and presence of fertilization. Similarly, the RDPI for the fertilization was the average of the relative distances between fertilization treatments in the absence and presence of the competition.

All statistical analyses were performed using the R statistical package version 3.2.4 (R Foundation for Statistical Computing). To compare morphological and functional traits among treatments and species, three‐way analyses of variance (ANOVA) were conducted using aov function. The measured traits were the dependent variable, and the fertilization treatment, competition treatment, species, and their interactions were independent variables. In order to interpret the species by treatment interactions, we conducted two additional analyses. First, to examine whether testing species exhibited differential trait values in each treatment, differences between species were evaluated for each treatment with post hoc Tukey mean comparison test (tukeyHSD function). To assess differential treatment effects among species, we conducted two‐way ANOVA for each species with environmental treatments and their interactions as independent variables. The total biomass, shoot biomass, and cross‐sectional area of achenes were log‐transformed to meet the normality assumption. To examine whether *Bidens* species showed differential phenotypic plasticity, one‐way ANOVA was conducted with the relative distances of a trait as dependent variable and species as independent variable. Differences between species were evaluated using *post hoc* Tukey mean comparison test.

## 3 RESULTS

### 3.1 Vegetative and functional traits

**FIGURE 2:**
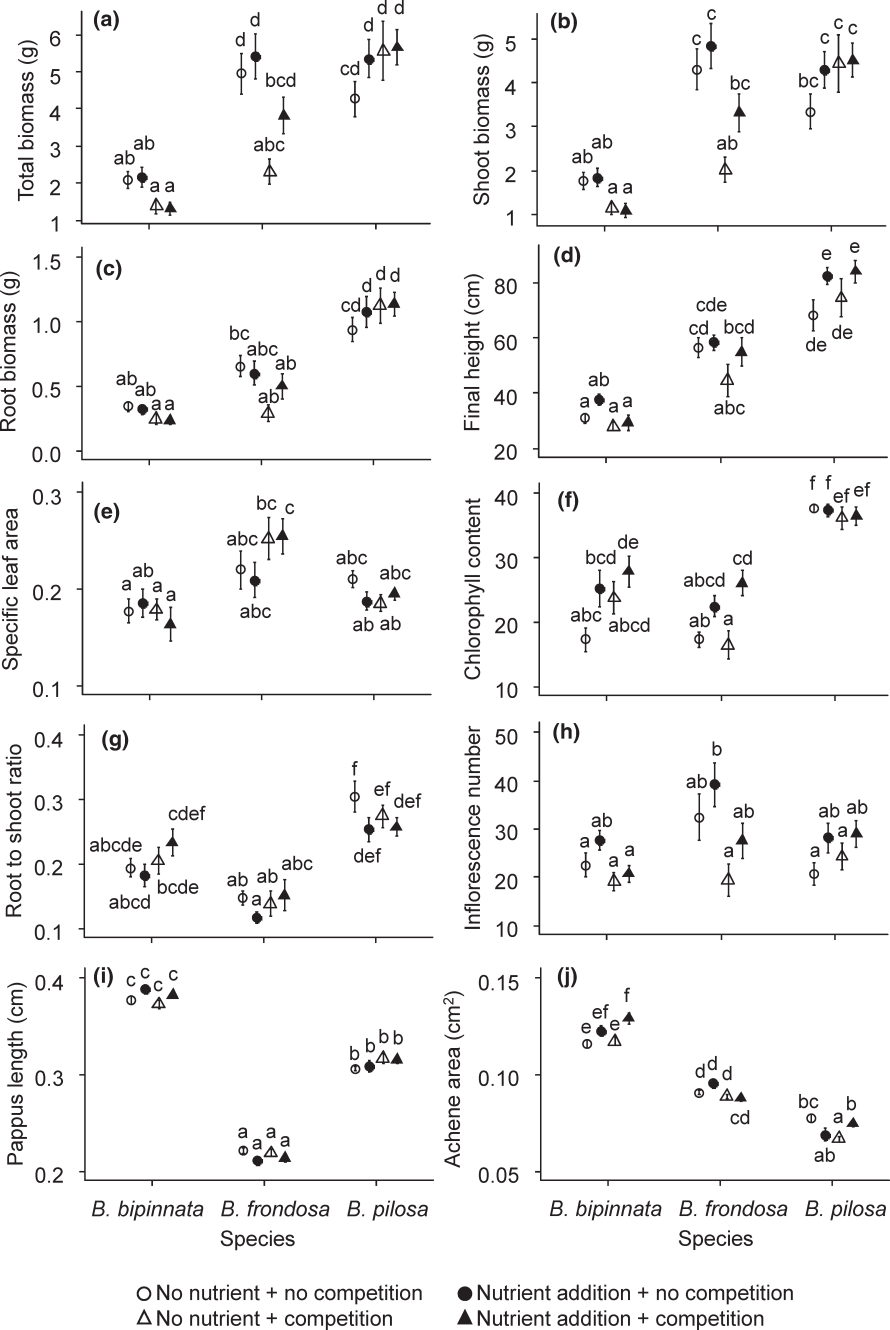
Effects of environmental treatments on the morphological and functional traits of three *Bidens* species. See Table 1 and Table S1 for the significance tests. Letters indicate statistically significant differences among species and treatments at the 0.05 level based on Tukey's adjustment. R/S ratio, root‐to‐shoot ratio, SLA, specific leaf area

When averaged across treatments, alien invasive *B. frondosa* and noninvasive *B. pilosa* produced more biomass than did native *B. bipinnata* (Table 1, Figure 2). All tested species increased their total biomass and shoot biomass in response to the fertilization treatment. In contrast, species responded to the competition treatment differently, as indicated by significant species by competition interactions (Table 1). Analyses of variance for each species revealed that total biomass and shoot biomass decreased under the competition treatment in *B. frondosa* (total biomass, *F*_1,54_ = 20.22, *p* < .001; shoot biomass, *F*_1,54_ = 22.04, *p* < .001) and *B. bipinnata* (total biomass, *F*_1,54_ = 13.16, *p* < .001; shoot biomass, *F*_1,54_ = 14.70, *p* < .001), but those of *B. pilosa* did not change (total biomass, *F*_1,55_ = 1.12, *p* = .29; shoot biomass, *F*_1,55_ = 1.17, *p* = .28) (Figure 2a,b, Table S1). When averaged across the fertilization treatment, the biomass of *B. frondosa* was smaller than that of *B. pilosa* in the competition treatment (adjusted *p* < .001), even though the two species had similar biomasses in the no‐competition treatment (adjusted *p* = 1.00). The responses of the final heights exhibited a similar pattern: *B. frondosa* (*F*_1,55_ = 5.34, *p* < .05) and *B. bipinnata* (*F*_1,47_ = 8.12, *p* < .01) presented decreased final heights in the competition treatment, while *B. pilosa* did not respond to the treatment (*F*_1,51_ = 0.35, *p* = .55). Across environmental treatments, *B. pilosa* exhibited a greater final stature than those of the other species (Figure 2d).

**TABLE 1:** Results of analysis of variance comparing morphological and functional traits among treatments and plant species

Traits	Species (*df* = 2)	Fert (*df* = 1)	Comp (*df* = 1)	Spc × Fert (*df* = 2)	Spc × Comp (*df* = 2)	Fert × Comp (*df* = 1)	Spc × Fert × Comp (*df* = 2)
Total biomass	74.30*	5.50*	16.80*	0.94	8.98*	0.37	0.10
Shoot biomass	70.84*	6.04*	17.51*	1.06	9.53*	0.16	0.95
Root biomass	90.80*	0.86	1.78	0.40	4.51*	0.22	1.38
Final height	111.72*	9.96*	6.15*	0.34	3.13*	0.02	1.30
SLA	16.41*	0.32	0.63	0.02	3.68*	0.19	0.97
Chlorophyll content	101.53*	17.30*	2.25	5.19*	2.29	0.04	1.13
R/S ratio	56.07*	1.27	0.99	1.45	1.52	3.52	0.02
Number of inflorescence	5.26*	10.00*	8.02*	0.44	5.47*	0.21	0.20
Pappus length	1835.10*	0.18	0.26	6.01*	2.23	0.00	0.26
Achene cross‐sectional area	535.25*	4.83*	1.73	4.16*	5.39*	6.75*	9.37*

Note

*F* ratios are given.

Abbreviations: Comp, competition; Fert, fertilization; R/S ratio, root‐to‐shoot ratio; SLA, specific leaf area.

* *p* < .05, **p < .01, ***p < .001.

Responses of other functional traits also differed among the investigated species. The SLA of *B. frondosa* increased in the competition treatment (*F*_1,51_ = 4.02, *p* = .05), but that of other species did not change (*B. bipinnata*, *F*_1,52_ = 0.54, *p* = .47; *B. pilosa*, *F*_1,55_ = 1.07, *p* = .31; Figure 2e). Consequently, differences in SLA between species were manifested only in the competition treatment (*B. frondosa* versus. *B. bipinnata*, adjusted *p* < .001; *B. frondosa* vs. *B. pilosa*, adjusted *p* < .001). Fertilization increased the chlorophyll content in *B. frondosa* (*F*_1,54_ = 16.78, *p* < .001) and *B. bipinnata* (*F*_1,35_ = 5.08, *p* < .05) but did not affect the chlorophyll content in *B. pilosa* (*F*_1,55_ = 0.00, *p* = .99) (Figure 2f). Even though chlorophyll contents of *B. frondosa* and *B. bipinnata* increased in response to fertilization, their chlorophyll contents (adjusted *p* < .001 and adjusted *p* < .001, respectively) were lower than those of *B. pilosa* under high‐nutrient conditions when averaged across the competition treatment. The R/S ratio of all three species did not respond to environmental treatments (Table 1, Figure 2g). *B. frondosa* exhibited a lower R/S ratio than did the other species.

**FIGURE 3:**
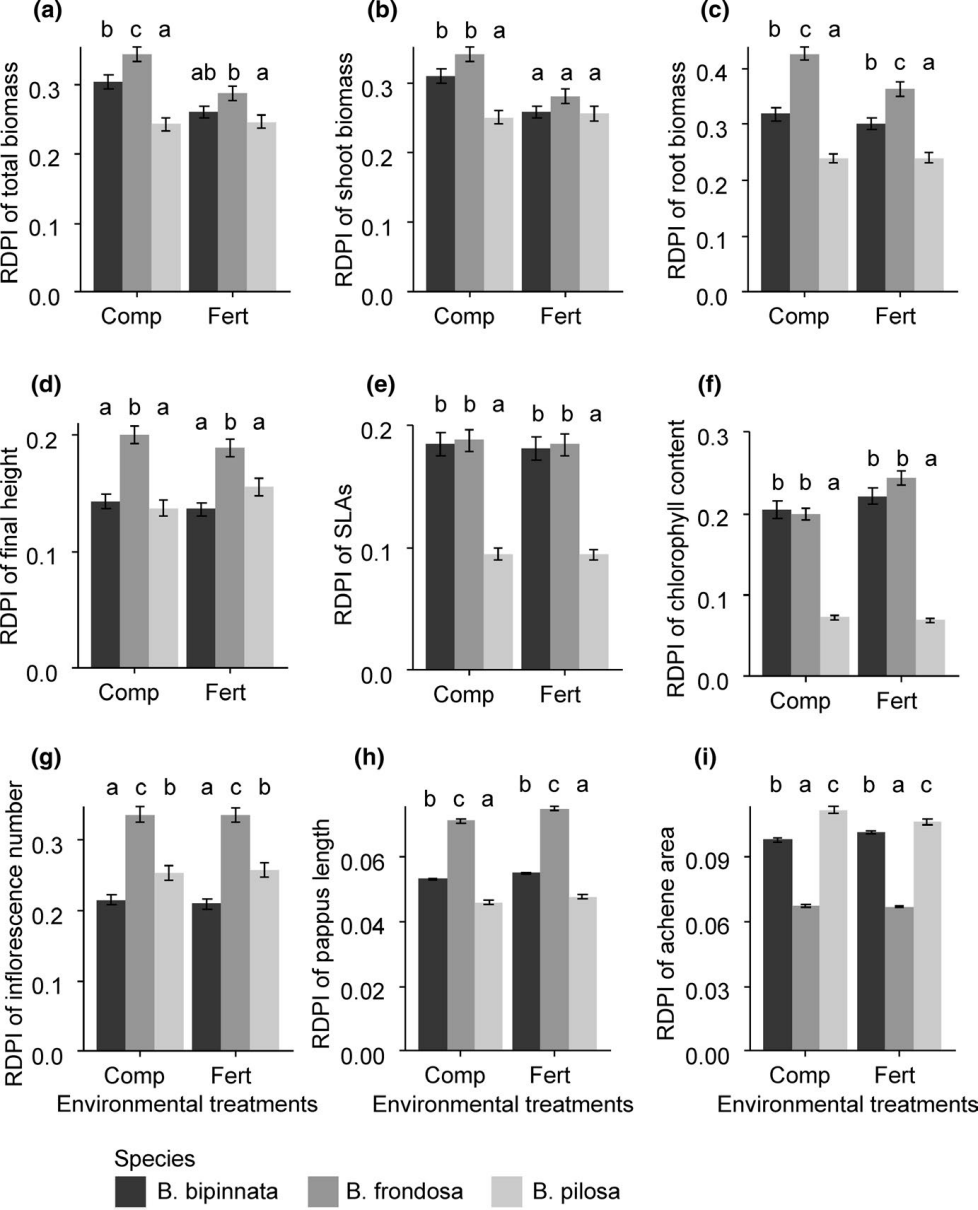
Relative distance plasticity index (RDPI) of morphological and functional traits of three *Bidens* species. Letters indicate statistically significant differences among species at the 0.05 level based on Tukey's adjustment. SLA, specific leaf area

In response to environmental treatments, testing *Bidens* species showed differential RDPIs in all measured vegetative and functional traits except the shoot biomass responding to the fertilization treatment (Figure 3). RDPI of *B. frondosa* was higher than those of noninvasive alien *B. pilosa* (Figure 3). *B. frondosa* exhibit higher RDPI than did native *B. bipinnata* in the total biomass, root biomass, and final height (Figure 3a,c,d), but RDPI of those two species was similar in the SLA and chlorophyll contents (Figure 3e,f).

### 3.2 Reproductive and achene traits

All tested species produced more inflorescences in response to the fertilization treatment. In contrast, the effect of competition on the inflorescence number differed among the tested species, as indicated by the statistically significant species by competition interaction (Table 1). The inflorescence number decreased under the competition treatment in *B. frondosa* (*F*_1,56_ = 9.03, *p* < .01) and *B. bipinnata* (total biomass, *F*_1,54_ = 6.29, *p* < .05), but that of *B. pilosa* did not change (*F*_1,55_ = 0.63, *p* = .43). The inflorescence number of *B. frondosa* had a higher RDPI value than those of the other two species (Figure 3g). When averaged across fertilization treatments, *B. frondosa* produced more inflorescences than did *B. bipinnata* (adjusted *p* < .01) and *B. pilosa* (adjusted *p* < .01) under the no‐competition treatment. In contrast, all three species produced a similar number of inflorescences when grown with competition (Figure 2h).

When averaged across fertilization and competition treatments, native *B. bipinnata* produced achene with a larger cross‐sectional area and longer pappus compared to those of other *Bidens* species (Table 1, Figure 2i,j). The achene morphology of the tested species responded to the fertilization treatment differently, as indicated by the significant species by fertilization interactions (Table 1). Under fertilization treatment, the pappus length of the achenes decreased in *B. frondosa* (*F*_1,246_ = 4.89, *p* < .05), increased in *B. bipinnata* (*F*_1,286_ = 6.65, *p* < .05), and did not change in *B. pilosa* (*F*_1,136_ = 0.01, *p* = .93). The achene area exhibited complicated responses to the experimental treatments. The achene area increased in *B. bipinnata* in response to the fertilization treatment (*F*_1,286_ = 13.81, *p* < .001) but did not respond to the competition treatment (*F*_1,286_ = 2.59, *p* = .11). The response to fertilization depended on the competition treatment in *B. frondosa* (*F*_1,246(fertilization × competition)_ = 5.15, *p* < .05) and *B. pilosa* (*F*_1,136 (fertilization × competition)_ = 13.72, *p* < .001). When plants were grown without competition, the achene area in *B. frondosa* increased, but *B. pilosa* decreased in achene area in response to the fertilization treatment (Figure 2j). In the competition treatment, the achene area of *B. frondosa* did not respond to the fertilization treatment, but that of *B. pilosa* increased (Figure 2j).

## 4 DISCUSSION

Invasive *B. frondosa* exhibited higher phenotypic plasticity in most measured traits compared to the noninvasive alien *B. pilosa* or native *B. bipinnata*. However, differential plastic responses to environmental treatments rarely altered the ranking of trait values among the three *Bidens* species, except for the number of inflorescences. The trait values and plasticity of the pappus length and achene size also differed among the investigated species and could potentially affect the dispersal efficiency.

### 4.1 Vegetative and functional traits

Massive biomass, tall stature at maturity, large SLA, and high chlorophyll content have been proposed as major attributes of invasive plant species (Divišek et al., 2018; Gallagher et al., 2014; van Kleunen et al., 2010; Leishman et al., 2007). These traits of testing *Bidens* species responded to the fertilization and competition treatments, but the responses differed among plant species (Figure 2). In particular, traits of noninvasive alien *B. pilosa* barely responded to the environmental treatments. Invasive *B. frondosa* exhibited higher RDPI than did *B. pilosa* (Figure 3). Even though environmental treatments affected the trait expression of both invasive *B. frondosa* and native *B. bipinnata*, *B. frondosa* had higher RDPI than those of native *B. bipinnata* did in the total biomass and final height (Figure 3). These results support the hypothesis that invasive plants tend to be more plastic than congenic native and noninvasive alien plants (Davidson et al., 2011).

Due to the higher plasticity, *B. frondosa* had more massive biomass after six weeks of growth and a greater final height than native *B. bipinnata*. Thus, the higher plasticity of *B. frondosa* regarding biomass and height likely confers a competitive advantage over native *B. bipinnata* (van Kleunen et al., 2015). Notably, differences in the biomass and final stature were more manifest when plants were grown without competition, indicating that the role of plasticity of *B. frondosa* would be of importance in disturbed habitats without other competing species.

The trait values of *B. frondosa* were similar to or even lower than those of noninvasive alien *B. pilosa,* though *B. frondosa* exhibited higher plasticity in most of the vegetative and functional traits. For instance, the plant height and biomass of *B. frondosa* increased when plants were grown without competition, but *B. frondosa* had a shorter stature and similar biomass relative to those of *B. pilosa* (Figure 2a,d). Leaf chlorophyll content showed a similar pattern, such that the chlorophyll content of *B. frondosa* increased in response to the nutrient addition but remained lower than that of *B. pilosa*. As van Kleunen et al. (2015) pointed out, the comparison of invasive versus noninvasive alien species is more appropriate for the identification of candidate traits determining invasive success than is the comparison of invasive versus native species. Our result suggests that the high plasticity of vegetative and functional traits likely play a limited role in the invasive success of alien *B. frondosa* even though it would contribute to the competitive advantages of invasive *B. frondosa* over native *B. bipinnata*.

The representative functional traits of invasive plant species include high SLAs and low R/S ratios, which are associated with greater resource capture abilities and faster growth rates (Gallagher et al., 2014; van Kleunen, Weber, et al., 2010; Leishman et al., 2007). The R/S ratios of the three investigated species were not plastic in response to environmental treatments, and invasive *B. frondosa* exhibited lower R/S ration than did native and alien noninvasive *Bidens* species across treatments. The homeostatic maintenance of a low R/S ratio would be beneficial particularly in a high‐nutrient environment (Gioria & Osborne, 2014); this likely explains the plastic increase of shoot biomass observed in *B. frondosa*. The three investigated species exhibited similar SLAs in the no‐competition treatment, while the SLA of *B. frondosa* was larger than that of the other species in the competition treatment. Since the effects of high SLA on the resource capture ability would be manifest in a high‐nutrient environment without competition (Gioria & Osborne, 2014), the contribution of SLA to the competitive ability of *B. frondosa* would be limited.

In summary, we could not find evidence that the plasticity of vegetative and functional traits contributes to invasive success. Instead, the absolute trait values rather than the plasticity of the R/S ratios likely contribute to invasive success (Godoy et al., 2012; Matzek, 2012).

### 4.2 Reproductive traits

Invasive *B. frondosa* produced more inflorescences than did native and noninvasive alien *Bidens* species, though this difference was manifested only when plants were grown without competition. Such a fecundity advantage would enhance the invasive success of *B. frondosa* and provide a competitive advantage over native *B. bipinnata*. *B. pilosa* and *B. bipinnata* exhibited a similar number of inflorescences across environmental treatments.

A previous meta‐analysis showed that the fitness components of invasive plants tended to be more plastic than those of native and noninvasive alien plants in response to nutrient availability (Davidson et al., 2011). Notably, this conclusion has been challenged, as the selection of native species for comparison might affect the results. For instance, alien and native weedy species exhibited similar responses to fertilization and competition (Dawson et al., 2012). Our results do support the hypothesis associating higher plasticity of fitness components with invasive species.

In this study, the total biomass and inflorescence number exhibited similar plasticity patterns in response to the experimental treatments. However, only the inflorescence number showed differential trait values among the tested species. The total biomass of *B. frondosa* and *B. pilosa* was similar even though the plasticity of *B. frondosa* was higher than that of *B. pilosa*. While fecundity is a more appropriate fitness measure than biomass for annual plant species (Crawley, 1997), total biomass has been widely used as a proxy of fitness based on the observation that biomass is highly correlated with reproductive output (Davidson et al., 2011; Dawson et al., 2012; Weiner et al., 2009). Our results suggested that the choice of fitness components might lead to contrasting conclusions, and diverse fitness components should be examined to evaluate the significance of traits associated with invasive success.

Since disturbance increases resource availability (Jauni et al., 2015), we expected that the fertilization and no‐competition treatments would likely affect the performance of the investigated plants in a similar manner. However, distinct effects were observed under these treatments; fertilization increased the inflorescence number of *B. pilosa,* but this effect was not detected under the no‐competition treatment (Figure 2h). In *B. frondosa*, the plastic inflorescence‐number response to the no‐competition treatment was higher than the response to the fertilization treatment. Therefore, the effects of disturbance may not be straightforward, as the removal of neighboring plants via disturbance would influence not only resource availability but also other biological interactions, such as the physical interference of root growth patterns (Padilla et al., 2013).

Invasive plants tend to have smaller seed sizes, potentially increasing their dispersal distance (Rejmánek & Richardson, 1996). In *Bidens* species, however, the length of pappi, in addition to the achene size, should be considered to evaluate the dispersal potential, since pappus length would affect the attachment and retention of achenes to animal furs (Couvreur et al., 2004; Kiviniemi & Telenius, 1998; Sorensen, 1986). Studies comparing seeds from multiple species found that seeds with more extensive spines were transported longer than seeds with short awns (Ansong & Pickering, 2016; Couvreur et al., 2004), indicating that a longer seed appendage likely has a positive effect on seed dispersal. In this study, the achenes of *B. pilosa* were smaller and featured longer pappi than those of *B. frondosa* (Figure 2i,j). Given that dispersal distance via adhesion has a positive relationship with the length of seed appendages but a negative correlation with the achene size, the noninvasive alien *B. pilosa* had a higher dispersal potential than did invasive alien *B. frondosa*. The achenes of *B. bipinnata* had the longest pappi and largest achene size among the three tested species, so it remains unclear whether it has a lower dispersal potential than the other species.

Long‐distance dispersal has been suggested as a key characteristic of the rapid range expansion of invasive species (Coutts et al., 2011; Rejmánek & Richardson, 1996). However, it should be noted that the fitness advantage of more distant dispersal depends on the ecological context. For instance, seeds that disperse further from maternal plants likely have a higher probability of facing novel environmental conditions. Because annual and biennial plants die after reproduction, no competition between maternal plants and offspring occurs. Thus, if the habitats of maternal plants provide favorable conditions for plant growth, annual plants would reap greater benefits from a short dispersal distance than from distant dispersal to novel environmental conditions. Through short‐distance dispersal, a population possibly accumulates large numbers of fruits that are sufficient to disperse into a novel habitat and establish there (Coutts et al., 2011; Hemrová et al., 2017). Since *B. frondosa* mainly occurs in agricultural fields with nutrient‐rich soil, a shorter dispersal distance and the accumulation of propagules would better promote its rapid expansion than would long‐distance dispersal.

The pappus length and achene size of the investigated species responded to nutrient addition differently. The pappus length of native *B. bipinnata* increased under the fertilization treatment, which likely increased its dispersal potential. In contrast, *B. frondosa* had shorter pappi and larger achene areas when grown in nutrient‐rich soil; therefore, its dispersal ability is expected to decrease under such conditions. The plasticity of the achene morphology in *B. frondosa* likely allows it to occupy favorable habitats, thereby increasing its population size and expansion rate.

## 5 CONCLUSIONS

Our results demonstrated that the invasive success of *B. frondosa* is likely attributable to its higher plasticity regarding the number of inflorescences. However, the phenotypic plasticity of most measured traits likely does not contribute to the invasive success of *B. frondosa*, since the trait values of *B. frondosa* were between those of noninvasive *B. pilosa* and native *B. bipinnata*. Invasive *B. frondosa* exhibited achene traits indicative of reduced dispersal ability compared with that of the noninvasive alien *B. pilosa*, in contrast with a previous hypothesis.

## CONFLICT OF INTEREST

The authors declare no conflict of interest.

## AUTHOR CONTRIBUTION

**Sunghyun Woo:** Data curation (lead); Formal analysis (lead); Investigation (lead); Methodology (lead); Writing‐original draft (lead). **Dongyeob Lee:** Data curation (lead); Formal analysis (lead); Investigation (equal); Visualization (equal). **Yong‐Chan Cho:** Conceptualization (equal); Funding acquisition (equal); Methodology (equal); Project administration (equal). **Sangsun Lee:** Data curation (equal); Formal analysis (equal); Investigation (equal); Visualization (equal). **Eunsuk Kim:** Conceptualization (lead); Data curation (lead); Formal analysis (lead); Funding acquisition (equal); Project administration (lead); Writing‐review & editing (lead).

## Supporting information

Table S1

## Data Availability

The dataset is available in Dryad https://doi.org/10.5061/dryad.0rxwdbrz3.
