# Supplementary material for: Differential responses to fertilization and competition among invasive, noninvasive alien, and native Bidens species
Source: Ecol Evol. 2020 Nov 24;11(1):516–25. doi: 10.1002/ece3.7071 (PMC7790614; doi:10.1002/ece3.7071)
Supplement: Supplementary file 1 — Table S1 [file ECE3-11-516-s001.docx]

Table S1. Results of analysis of variance testing effects of treatments on morphological and functional traits in each species. Traits with significant species by treatment effect in Table 1 were analyzed. F ratios are given. Fert = fertilization, Comp = competition, R/S ratio = root to shoot ratio, SLA = specific leaf area. ** P* < 0.05, *** P* < 0.01, **** P* < 0.001.

| Species | Traits | Fert  (d.f. = 1) | Comp  (d.f. = 1) | Fert × Comp (d.f. = 1) |
| --- | --- | --- | --- | --- |
| *B. bipinnata* | Total biomass | 0.10 | 13.16*** | 0.01 |
|  | Shoot biomass | 0.08 | 14.70*** | 0.00 |
|  | Root biomass | 0.19 | 6.27* | 0.02 |
|  | Final height | 2.84 | 8.12** | 1.78 |
|  | SLA | 0.05 | 0.54 | 0.68 |
|  | Chlorophyll content | 5.08* | 2.98 | 0.50 |
|  | Number of inflorescence | 2.75 | 6.29* | 0.80 |
|  | Pappus length | 6.65* | 1.56 | 0.05 |
|  | Achene cross sectional area | 13.81*** | 2.59 | 0.80 |
| *B. frondosa* | Total biomass | 5.14* | 20.22*** | 2.13 |
|  | Shoot biomass | 5.71* | 22.04*** | 1.85 |
|  | Root biomass | 0.89 | 7.28** | 2.33 |
|  | Final height | 2.25 | 5.34* | 1.02 |
|  | SLA | 0.05 | 4.02 | 0.12 |
|  | Chlorophyll content | 16.78*** | 0.63 | 1.49 |
|  | Number of inflorescence | 3.36 | 9.03** | 0.04 |
|  | Pappus length | 4.89* | 0.01 | 0.49 |
|  | Achene cross sectional area | 1.89 | 12.90*** | 5.15* |
| *B. pilosa* | Total biomass | 2.22 | 1.12 | 0.24 |
|  | Shoot biomass | 2.60 | 1.17 | 0.31 |
|  | Root biomass | 0.41 | 1.25 | 0.35 |
|  | Final height | 6.42* | 0.35 | 0.17 |
|  | SLA | 0.68 | 1.07 | 3.75 |
|  | Chlorophyll content | 0.00 | 0.96 | 0.07 |
|  | Number of inflorescence | 4.94* | 0.63 | 0.26 |
|  | Pappus length | 0.01 | 3.50 | 0.12 |
|  | Achene cross sectional area | 0.12 | 1.04 | 13.72*** |
